# Structural Diversity: A Multi-dimensional Approach to Assess Recreational Services in Urban Parks

**DOI:** 10.1007/s13280-014-0508-9

**Published:** 2014-04-17

**Authors:** Annette Voigt, Nadja Kabisch, Daniel Wurster, Dagmar Haase, Jürgen Breuste

**Affiliations:** 1 Department of Geography and Geology, University Salzburg, Hellbrunnerstr. 34, 5020 Salzburg, Austria; 2 Institute of Geography, Humboldt University Berlin, Unter den Linden 6, 10099 Berlin, Germany; 3 Department of Urban and Environmental Sociology, Helmholtz Centre for Environmental Research – UFZ, 04318 Leipzig, Germany; 4 Department of Computational Landscape Ecology, Helmholtz Centre for Environmental Research – UFZ, 04318 Leipzig, Germany

**Keywords:** Urban parks, Assessment, Structural diversity, Recreation

## Abstract

Urban green spaces provide important recreational services for urban residents. In general, when park visitors enjoy “the green,” they are in actuality appreciating a mix of biotic, abiotic, and man-made park infrastructure elements and qualities. We argue that these three dimensions of structural diversity have an influence on how people use and value urban parks. We present a straightforward approach for assessing urban parks that combines multi-dimensional landscape mapping and questionnaire surveys. We discuss the method as well the results from its application to differently sized parks in Berlin and Salzburg.

## Introduction

Both quantity and quality of urban parks are increasingly recognized as important for the quality of urban life regarding a wide range of benefits and ecosystem services (e.g., Burgess et al. 1988; Chiesura 2004; Breuste et al. 2013; see systematic review Konijnendijk et al. 2013). Public urban parks are specifically designated for diverse active and passive recreation activities, mostly accessible free of charge and without long distance traveling (Wolf and Appel-Kummer 2009). They are mostly designed for different demands as multifunctional recreational areas (Maruani and Amit-Cohen 2007) and have to meet the diverse requirements of contemporary society. However, there is a lack of knowledge regarding the relationship between park characteristics and visitors’ activities and demands. Which properties and elements are attracting people and what is the quality of recreational experience is offered by them? How important is the diversity of park features for the respective activity and experience? Are more people attracted by a high diversity of natural features or by a big number of sports facilities and other man-made infrastructure? In the context of urban park development, answers to these questions may help explain the differences regarding demand and supply of recreational services, and therefore provide guidance for how to better plan and manage parks with people and their diverse interests in mind.

We present and discuss an integrative method that combines multi-dimensional mapping of urban parks’ structural diversity regarding biotic features, abiotic site conditions, and man-made infrastructure with users’ activities and their demand on park characteristics. Furthermore, we show the results of two small scale applications of this method in six differently sized urban parks and discuss whether and how parks’ various physical properties influence visitors’ recreational activities.

### Research on the Demand and Supply of Urban Parks’ Recreational Services

We refer to recreation as an experience or activity of leisure, an outcome of discretionary time being free from prior commitments to physiologic or social needs (Yukic 1970, p. 5). Our focus is limited to outdoor recreation in urban parks, including both active physical exercises such as walking, biking, or jogging as well as passive relaxation activities such as sunbathing, reading, or nature observation. Therefore, urban parks offer opportunities for improving physical health and psychological well-being, the latter potentially also through nature experience, social contacts, and relaxation. The therapeutic and recreational effect of the park depends not only on the park’s properties, but of the person concerned, his/her current situation or well-being as well as side conditions such as the weather. Nevertheless, there is a consensus that nature in most cases has positive effects on well-being (Kaplan and Kaplan 1989; Ulrich et al. 1991).

Research mostly either places the park visitors or the park’s facilities and properties in the foreground, but these two perspectives were quite rarely joined systematically. Social research focuses on visitors or local residents, their demands on and the perception of the respective park, their leisure activities there as well as their experiences, satisfaction, and conflicts (e.g., Loukaitou-Sideris 1995; Tyrväinen et al. 2007). For example, Chiesura (2004) asked park visitors in Amsterdam for their intended leisure activities, their nature experience, and its importance for well-being. Oguz (2000) focused on the relationship of visitors’ personal data with their activities and satisfaction and found that the use of each park has its own characteristics. An important research subjects are the use patterns, preferences, and perception of user subgroups distinct regarding cultural background, ethnicity, age, gender, or phase of life (e.g., Loukaitou-Sideris 1995; Gobster 2002; Low et al. 2005; Shores and West 2008). However, most of this research disregarded the supply side. Studies regarding supply concentrate on objective measures and/or on the residents’ self-reported perceptions of park qualities seen as important for encouraging park use such as size, maintenance condition, safety, and proximity (Loukaitou-Sideris 1995; Giles-Corti et al. 2005; Kaczynski et al. 2008; Cohen et al. 2010). Also, properties that diminish or prevent visits such as reduced safety (Schroeder and Anderson 1984; Burgess et al. 1988; Bixler and Floyd 1997) and poor maintenance (Gobster 2002; McCormack et al. 2010) are highlighted. While most prior research focused on single attributes, Gobster and Westphal (2004) pointed to the multi-dimensionality by the example of the Chicago River greenway. They identified six “human dimensions”—cleanliness, naturalness, esthetics, safety, access, and appropriateness of development—which together form a core set of concerns relating to how people perceive and use the greenway. McCormack et al. (2010) summarized in a review that besides safety, esthetics, maintenance, and proximity, particular facilities (playgrounds, sport courts, trails) are important for physical activities. These multi-dimensional findings meet our interest in how the park’s physical features influence people’s use and valuation. In this respect, most studies, especially in health research, analyze how man-made recreational facilities promote physical activity (e.g., Cohen et al. 2007; Floyd et al. 2008; Shores and West 2008; see also reviews by Kaczynski and Henderson 2008; McCormack et al. 2010). Few surveys include the influence of some biotic features such as wooded areas or meadows (Kaczynski et al. 2008) or flowerbeds (Poje et al. 2013). However, most of these studies are limited to facilities for physical activities and do not cover passive, contemplative, or relaxing recreation. In addition, few studies examine the perception and valuation of biotic features and abiotic side conditions although their influence on well-being regarding physical, sensory, or psychological functions as well as esthetic and symbolic values is often postulated (e.g., Smardon 1988). More (1985) showed that people’s use of public parks can be influenced by vegetation types; for example, grass is positively correlated with sleeping, indulging, eating, playing, and reading. According to Fuller et al. (2007), visitors’ psychological well-being is positively correlated with species richness and habitat diversity; in contrast Dallimer et al. (2012) found no consistent relationship between actual biodiversity and well-being, but a positive link between the perceived level of biodiversity and well-being.

The literature review shows that studies hardly examine the connection of supply and demand of recreational services in urban parks, but when they do so, they highlight the connection of facilities and physical activities only. Few studies regard natural features. Consequently, there is a research gap in reference to the relation between biotic features, abiotic side conditions, man-made infrastructure, and the visitors’ demand as well as the influence of park characteristics on active and passive leisure activities. In the following, we present a feasible approach linking the mapping of data concerning the park’s multi-dimensional structural diversity and a questionnaire on visitors’ activities and their self-reported importance of specific characteristics for their well-being. To test this method, we used a sample of six urban parks in two European cities and linked the results.

## Materials and Methods

### Study Sites

Surveys were conducted in public parks in Berlin, Germany, and Salzburg, Austria. These two cities vary greatly in size and population as well as in surrounding landscape and therefore provide different conditions to test the method.

Berlin is the largest German city (89 174 ha) with more than 3.5 million inhabitants. About 14 % of the city area is public green, 18 % forest, and 7 % water bodies (Senatsverwaltung für Stadtentwicklung und Umwelt 2012). We survey four small parks situated in central dense built-up districts. The Köllnischer Park (KP) is the smallest green space (about 1 ha) in our analysis. It is located near the Spree River and contains some historical buildings. The Engelbecken (EB) is part of the green corridor Luisenstädtischer Kanal, a former canal, and therefore is lower than the surrounding area. Its main feature is a large rectangular water basin. It is protected under historic preservation laws. The Mariannenplatz with its adjacent green space (MP, ca. 8 ha) is part of an intra-urban green corridor. The Carl-Herz-Ufer (CHU, 1.8 ha) is a narrow park accompanying an urban canal.

Salzburg, the fourth-largest city in Austria (150 000 inhabitants) is located at the Alps’ northern fringe. Its administrative area covers 6567 ha, about 58 % is green and blue (Magistrat der Stadt Salzburg 2012). Green areas (including agricultural land and forests) are legally protected. The two parks selected, Lehner Park (LP) and Hans-Donnenberg-Park (DP), differ in size, structural diversity, and the built-up density of the surrounding urban landscape. The DP (7 ha) is located within a less densely built-up residential area. It was built around 1965 as the extension of an old garden and a tree nursery and is partially surrounded by areas used for urban agriculture and recreational purposes. The LP (3 ha) is located within Salzburg’s most densely built-up district, next to the Salzach River and its accompanying walk- and bikeway.

The parks in the two cities are differently sized. While the selected parks in Berlin are rather small (1–2 ha except the Mariannenplatz with its adjacent green space), the two parks in Salzburg are comparatively large (3–7 ha). The selection of differently sized parks is based on the assumption that larger parks may be more used by residents than smaller parks (Schipperijn et al. 2010) because of the higher structural diversity commonly found in larger parks. We will also refer to the issue of park size in the discussion of the results.

### Mapping Tool for Urban Parks’ Multi-dimensional Structural Diversity

For urban parks, instruments to audit recreational facilities for physical activities have been developed. Some instruments focus on condition and maintenance (e.g., Cavnar et al. 2004); others on the multitude (e.g., Giles-Corti et al. 2005). Saelens et al. (2006) developed a comprehensive instrument (EAPRS) for the assessment of public recreation spaces with an emphasis on the functionality of physical elements for active use that was also used repeatedly (e.g., Kaczynski et al. 2008; Van Dyck et al. 2013). However, most instruments regard the facilities for physical activities only, thus overlooking biotic and abiotic conditions, aspects that may promote other recreation forms. In contrast, the assessment of landscape’s nature-related diversity for its adequacy for recreation (e.g., V-value-method by Kiemstedt 1967; Zube et al. 1975) is not adaptable to urban parks because it does not incorporate man-made infrastructure.

In contrast to these instruments, we developed a mapping tool referring to urban parks’ multi-dimensional structural diversity defined as diversity which includes biotic features, abiotic site conditions, and infrastructure facilities. We assumed that each of these three dimensions affects park visitors’ evaluation and activities. Instead of focusing on single element such as species, we used a structural level regarding visually dominant features such as meadows, lawns, and groups and rows of trees. There are two reasons for choosing this structural level: it is easy to apply and enables the comparison of different parks in different ecological zones. In addition, people generally have poor species identification skills (Dallimer et al. 2012), and we assumed that they perceive biotic features on a more structural level.

**Fig. 1:**
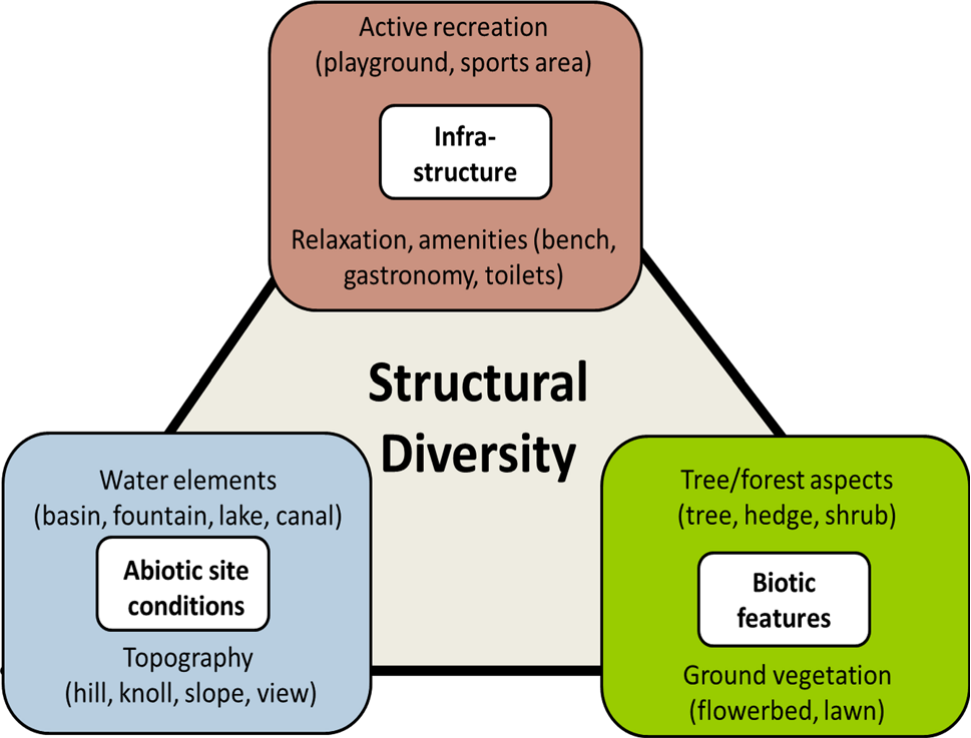
Conceptual interpretation of structural diversity of urban parks

Figure 1 visualizes the conceptual approach of multi-dimensional structural diversity. Each of the dimensions is separated into two main categories; for example, for the dimension *biotic features* the categories are “tree/forest aspects” and “ground vegetation”, each including various elements (see also Table 1). In the subdivisions, we tried to reach the corresponding level of detail for all three dimensions. The selection of elements was based on a comprehensive literature review on park research and mapping instruments (e.g., Hemphill et al. 2004; Cavnar et al. 2004; Giles-Corti et al. 2005; Saelens et al. 2006), on guidelines for urban biotope mapping used in Germany (e.g., Senckenberg 2007) as well as on our former experiences in park analysis and mapping (Rall and Haase 2011). “Biotic features” contain tree/forest aspects and ground vegetation in respect to both (semi-)natural as well as ornamental vegetation. The first category includes solitary trees as well as group/row of trees taking into account the age/size. We considered also whether the average of the tree species diversity is more than five species on half a hectare. Other elements are hedge, bush, and natural-like, dense wooded area with underbrush. In the category “ground vegetation”, one can note whether there is diverse spontaneous ground vegetation such as herbs or tree seedlings as well as diversity at the water’s edge regarding wetland plants. Further elements are grassed areas of extensive use and management (meadow), lawns with intensive management and use (e.g., for ball games or sunbathing), and flowerbeds.

**Table 1:** Overview of the dimensions, categories, elements, and results (expressed as “1” and “0”) of the structural diversity mapping in the urban parks. The normalized value for each dimension for the parks is given in the lines below the respective dimension. For example, if you are considering “tree/forest aspects” and “ground vegetation” for KP as the two elements within biotic features to normalize, then you get 0.51 for biotic features with the equation ((5/8) + (2/5))/2 = 0.51)

Dimension	Category	Element	KP	EB	CHU	MP	DP	LP
Biotic features	Trees/forest aspects	Tree species diversity (>5 species/0.5 ha)	0	0	0	0	1	0
		Solitary trees big/old	1	1	1	1	1	1
		Solitary trees small/young	1	0	0	0	1	0
		Group of trees	0	0	1	1	1	1
		Row of trees/tree-lined path	1	1	1	1	1	0
		Hedge (trimmed or untrimmed)	1	1	0	1	0	1
		Shrub	1	1	1	1	1	1
		Natural-like, Dense-wooded area (trees, underbrush)	0	0	1	0	1	0
	Ground vegetation	Diverse spontaneous vegetation (herbs, tree seedlings)	0	0	1	0	1	0
		Diverse water edge (wetland plants)	0	0	0	0	1	0
		Grassed areas/lawn extensive (meadow area)	0	0	1	0	1	1
		Lawn intensive (open access)	1	1	0	1	1	1
		Flowerbed	1	1	0	1	0	1
Normalized value for: biotic features			0.51	0.45	0.51	0.51	0.84	0.55
Abiotic site conditions	Water elements	Water basin	0	1	0	0	0	0
		Fountain	0	1	1	1	0	0
		Natural or near-natural lake/pond	0	0	0	0	1	0
		Flowing watercourse in the park	0	0	1	0	1	0
		(Visual) dominant water element in neighborhood	1	0	1	0	0	1
		Good/direct access to water edge	0	0	1	0	0	0
	Topography	Attractive view	0	1	1	0	1	0
		Hill/knoll	0	0	0	0	0	1
		Slope	0	0	1	0	1	0
		Artificial surface lowering or elevation (“stairs”)	0	1	1	1	0	1
Normalized value for: abiotic conditions			0.08	0.42	0.71	0.21	0.42	0.33
Infrastructure	Active recreation	Distinct bicycle path	0	0	0	0	1	0
		Designated sport or athletic fields (e.g., with goals for football)	0	0	0	1	1	1
		Street or basketball court	0	0	0	1	1	1
		Table tennis table	0	0	0	1	1	0
		Large/diverse playground for kids (>5 elements)	1	0	1	1	1	1
		Dog park	0	0	0	0	1	0
	Relaxation/amenities	Sitting features: Bench, seat wall	1	1	1	1	1	1
		Picnic table, shelter, pavilions	0	0	0	1	0	1
		Historical, artistic, or educational landmark	1	1	0	1	1	1
		Animal compound/petting zoo	1	0	0	0	0	0
		Gastronomy	0	1	1	0	0	0
		Drinking fountain	0	0	0	0	1	1
		Public sanitation	1	0	0	1	0	1
		Lighting (of main paths)	0	1	1	1	1	1
Normalized value for: infrastructure			0.33	0.25	0.27	0.65	0.75	0.63

The second dimension—abiotic site conditions—refers to the category of natural or man-made water elements such as water basin, fountain, natural or near-natural lake or pond, and flowing watercourse. In addition, we regarded whether there is a visually dominant water element in the park neighborhood and whether the given water elements are directly accessible. The category “topography” includes the elements hill or knoll, slope, artificial surface lowering or elevation (“stairs”), and dominant stone or rock formation. Here, the quality of view from the park onto the surroundings is also included. Finally, the third dimension is infrastructure, which defines park facilities for physical active recreation, as well as amenities and facilities for physical passive relaxation. Elements for exercise are distinct bicycle paths within the park, designated athletic field (e.g., with goals), street-/basketball court, and ping-pong table. We also include playground and dog park. Mapped amenities and facilities for passive relaxation include anything constructed for sitting; table, shelter, and pavilion for picnics; historic, artistic, or educational landmark; animal enclosure or petting zoo; any kind of gastronomy; drinking fountain; public restroom. We also consider whether the main paths are lighted.

### Mapping and Evaluation of Structural Diversity

Two skilled collaborators mapped the parks in September 2013. They recorded only the presence of the particular property or component, not the number or quality. Therefore, a park with several football fields does not differ from a park with only one. In addition, they recorded components according to their visual dominance. That means that, for example, one young solitary tree standing somewhere off site has not been counted as it has no influence on the visual ensemble or characteristic of the park.

We calculated a coefficient using a simple additive procedure of all assessed characteristics and components. Values were then normalized by the total number of possible elements of each sub-category to make the results comparable. Finally, the mean value of the two sub-categories is shown as the value for total structural diversity in the biotic elements, abiotic site conditions, and infrastructure elements. A calculation example is given in Table 1.

### Questionnaire Surveys Assessing Visitors’ Activities and Demands

To identify visitors’ demands, we conducted face-to-face interviews. The interviews were set up after smaller pre-test studies with randomly selected visitors on site as well as with students who we debriefed, that is we posed structured follow-up questions to elicit qualitative information about their interpretations of questions. This helped us to improve the wording for making sure that the questions were eliciting the kinds of responses intended. For example, we selected neutral terms for describing park characteristics (such as “lively going on” instead of “rather crowded situation”). Finally, questionnaires were distributed on both weekends and weekdays during different hours of the day to randomly selected respondents at different park stations. Approached people were first informed about the survey’s objective and answering procedure. Those willing to participate were invited to fill in the questionnaire together with the interviewer (students trained in the procedures and etiquette of conducting the survey). In the parks, the refusal rate varied (20–35 %).

The questionnaire addressed a broad range of issues, but for the purpose of this paper, the analysis is limited to two questions. First, we asked, “What activities are you undertaking in this green space today?” and allow the respondent 3 replies. The second question aimed at the importance of park characteristics for the well-being of visitors. We asked, “How important are the following park characteristics for your well-being today: accessibility, attractive plants and wildlife (biotic features), facilities for relaxation (passive recreation), and facilities for sport and play (active recreation)?” In Salzburg, we also asked about landscape beauty, view, naturalness, and tranquility or lively going on. In Berlin, we additionally asked about shaded areas and proximity to water because of the dominant water elements in some of the parks. We request that interviewees rate the importance they place on these features on a Likert-scale ranging from 1 (not important) to 5 (very important).

### Observation Protocols

Some people refused to participate in the survey due to participation in sports, group activities, or playing with children or because of deficient language skills (immigrants or tourists), so these groups are under-represented in the findings. Therefore, we made additional observation protocols on users’ activities. For this, a quantitative count of the number of different activities was carried out using a standardized observation protocol. To perform the counting, trained observers walked a fixed route (40 min) through the study areas and counted all observed activities.

## Results

### Structural Park Diversity in Salzburg and Berlin

**Fig. 2:**
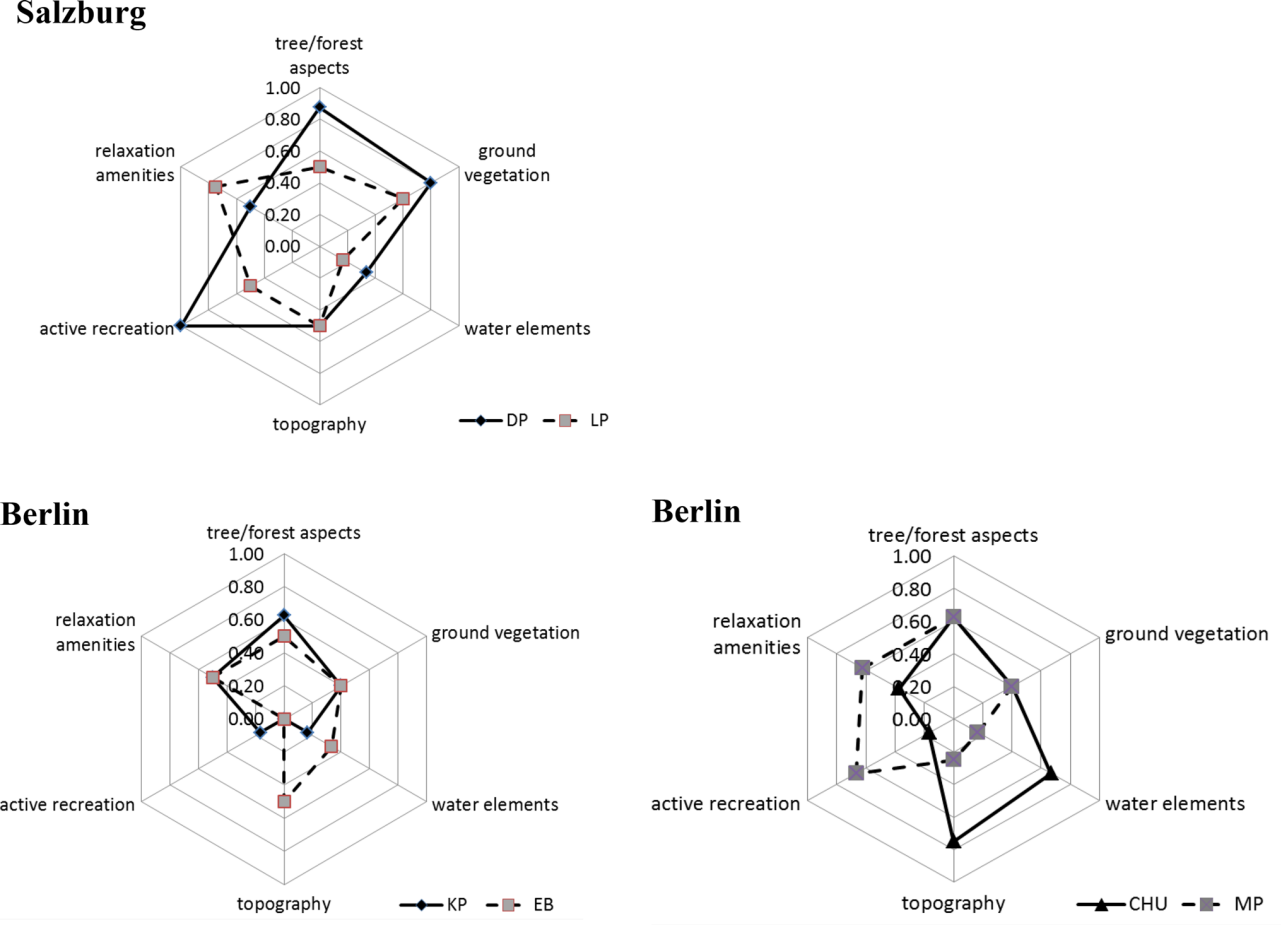
Values for the components of biotic elements, abiotic site conditions, and infrastructure elements for parks in Salzburg and Berlin

The mapping results are shown in Table 2 and Fig. 2. In Salzburg, the Hans-Donnenberg Park (DP) is characterized by a natural slope. In the upper part, a number of infrastructural facilities for physical activities exist, such as a diverse playground, various sport fields and lawns, drinking water, and benches. The main paths are lighted. There is a beautiful view to the Alps from the upper part. In the lower part, there is a dog park, extensively managed meadows, ponds with diverse wetland vegetation, a little stream, and zones of dense woodland with shrubs and trees of different ages. We found more than 50 tree species in DP and a great diversity of spontaneous herbaceous plants. The structural diversity mapping resulted, thus, in high values for trees and forest aspects (0.88) and also relatively high values for ground vegetation (0.80). In total, the value for biotic features is high (0.84), while for infrastructure facilities it is slightly lower (0.75) and the value for abiotic site conditions is low (0.42). The second assessed park in Salzburg, the Lehner Park (LP) contains a playground, a streetball court, benches, picnic tables, drinking water, and a public restroom. The main paths are lighted. The general diversity value for infrastructural elements, thus, reached a total value of 0.63. Differentiated by the two categories active and passive recreation, the values are 0.50 and 0.75, respectively. At the western edge, a little semi-natural meadow prevents intensive use. The total value for the biotic features is 0.55. The LP has a visually dominant water element in the neighborhood, the Salzach River, and a little hill. Altogether, it resulted in a value of 0.33 for abiotic site conditions.

**Table 2:** Assessment of structural diversity, relative shares of park characteristics, and visitors’ activities

	Berlin				Salzburg	
	KP	EB	CHU	MP	DP	LP
Structural diversity						
Tree/forest elements	±	−	±	±	+++	−
Ground vegetation	−	−	−	−	+++	+
Water elements	−	±	+++	−	±	−
Topography	−−	±	++	−	±	±
Active infrastr.	−	−−	−	+	+++	±
Passive infrastr.	±	±	−−	+	±	+++
Park characteristics						
Attractive wildlife	0.68	0.70	0.70	0.68	0.56	0.71
Attractive plants	0.75	**0.83**	0.79	0.67	0.67	0.73
Naturalness	n.a.	n.a.	n.a.	n.a.	**0.93**	**0.87**
Beauty of landscape	n.a.	n.a.	n.a.	n.a.	**0.76**	0.75
Good view	n.a.	n.a.	n.a.	n.a.	0.54	0.59
Tranquility	n.a.	n.a.	n.a.	n.a.	**0.79**	**0.79**
Facilities for relaxation	**0.80**	0.80	0.88	**0.88**	0.70	0.68
Facilities for sport and play	n.a.	n.a.	n.a.	n.a.	0.58	0.72
Access	**0.87**	**0.86**	**0.82**	**0.90**	**0.76**	**0.76**
Proximity to water	0.69	**0.86**	**0.86**	0.64	n.a.	n.a.
Shaded areas	**0.85**	0.77	**0.80**	**0.86**	n.a.	n.a.
Activities (%)						
Active/sport	6.82	2.04	10.42	4.00	**75.00**	47.00
Passive/relax	**93.18**	**97.96**	**89.58**	**96.00**	25.00	**53.00**

Structural diversity values range from low to high represented with—as low and +++ as high (in each category values between the mean and 0.5 × standard deviation are presented as ±; values < or >0.5 × standard deviation around the mean are presented as − or +; values < or >1 standard deviation around the mean are presented as ++ or − and values < or >1.5 × standard deviation around the mean are presented as +++ or −−−). Park characteristics are normalized to 0–1, ranging from “not important” to “very important”

*n.a.* not assessed in this park

In Berlin, the Engelbecken (EB) is dominated by a rectangular pool that is part of a former canal. It is encircled by paths with benches. There are a café, an additional fountain as well as intensively managed lawns and intricate flowerbeds with sundry perennial herbaceous plants. There are no further active recreation facilities so that relaxation amenities dominate (value 0.50). In sum, the infrastructure is rather low at 0.25 because of the lacking active-oriented elements (Fig. 2). Although the EB is intensively managed with nice flowerbeds, shrubs, and hedges, the number of trees and tree diversity is rather low. Thus, the value for biotic elements is 0.45. The park is rather flat, but due to the dominant water element, the artificial surface, and the overall nice view of the historic area, the abiotic site conditions are valued at 0.42. The third assessed park in Berlin—Köllnischer Park (KP)—has a large playground, benches, a public toilet and, as an attraction, a bear enclosure. As the only park of the four assessed, the KP has no lighting of the main paths. In effect, the value for the infrastructure dimension is 0.33. The KP also has a number of young and old solitary trees and simple flowerbeds. The biotic features result in a value of 0.51. The fourth park—Mariannenplatz (MP)—contains intensively used and managed lawns, shrubs that border the street, and a number of large old trees. Further, there are benches, public restrooms, sculptures, and a paved space with a fountain. There is a large playground, a table tennis area as well as a soccer field and basketball court in the adjacent green space. The value for infrastructure elements is accordingly high with 0.65. The value for the biological dimension is 0.51 because of additional solitary big old trees, groups of old trees, some shrubs, and hedges. No young solitary trees or groups of young trees could be found. Finally, the Carl–Herz–Ufer (CHU) is a green space in close proximity to an old canal. It contains old trees along the canal and some densely wooded natural areas. There are some lawn areas but no hedge or flowerbed. The value for the biotic diversity is 0.51 while it is highest for abiotic site conditions (0.71). Beside the canal, which can be directly accessed, there is also a fountain. There are benches, a café, and a large playground but no public restrooms or spacious athletic field in this area. The value for the infrastructure dimension is rather low at 0.27.

Whereas the parks differ widely regarding biotic features and abiotic site conditions, the differences in the number of facilities for active recreation and relaxation are lower. The Austrian DP has the highest diversity of biotic features while Berlin’s CHU shows the highest abiotic diversity. The DP seems to be the most diverse park because its infrastructure diversity is also very high. In contrast, the smaller parks in Berlin have only one dominant diversity dimension—such as the infrastructure dimension in the MP or the aforementioned abiotic values for the CHU—or have generally lower diversity values.

### Activities of Park Visitors

**Fig. 3:**
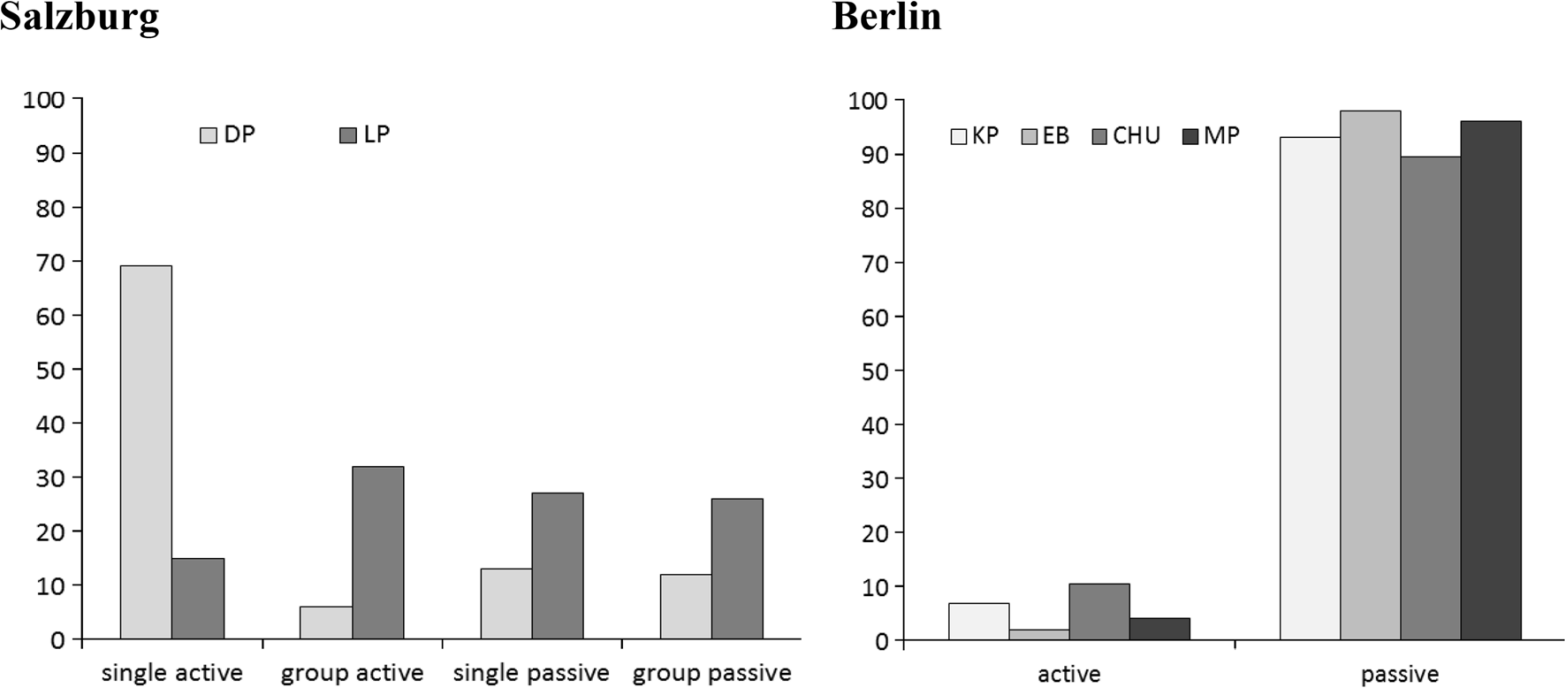
Visitors’ activities in Salzburg’s and Berlin’s urban parks (DP: *n* = 107; LP: *n* = 265; KP: *n* = 48; EB: *n* = 49; CHU: *n* = 49; MP: *n* = 51). *Note*: Only in Salzburg, activities were classified into single and group activities and data from observation protocols were included

For the evaluation of the activities in the two parks in Salzburg, results of surveys and observation protocols on users’ activities have been combined. Since park visitors normally engage in various activities during their stay, multiple answers and counts were allowed. This led to a total number of 107 answers and counts in DP and 265 in LP. For practical reasons, we grouped the answers: “Active single” includes walking, walking the dog as well as jogging and other single sporting activities; “active group” pools activities such as playing with children and group sports. “Single passive” includes watching other people, reading, sunbathing, enjoyment of flora and fauna whilst “passive group” includes communicating with other people or picnicking. In Berlin, the activities were grouped into passive activities and physical active exercises; in other words, no distinction was made between group and single. The results are shown in Fig. 3.

The results in Salzburg show that in DP most of the people do single active exercises. By contrast, more than half of the people surveyed in LP rather act passively no matter whether single or group activities. In Berlin, nearly all people do passive activities and rather relax on benches, sunbathe or watch their children playing.

### The Importance of Park Characteristics for Well-Being by Visitors

**Fig. 4:**
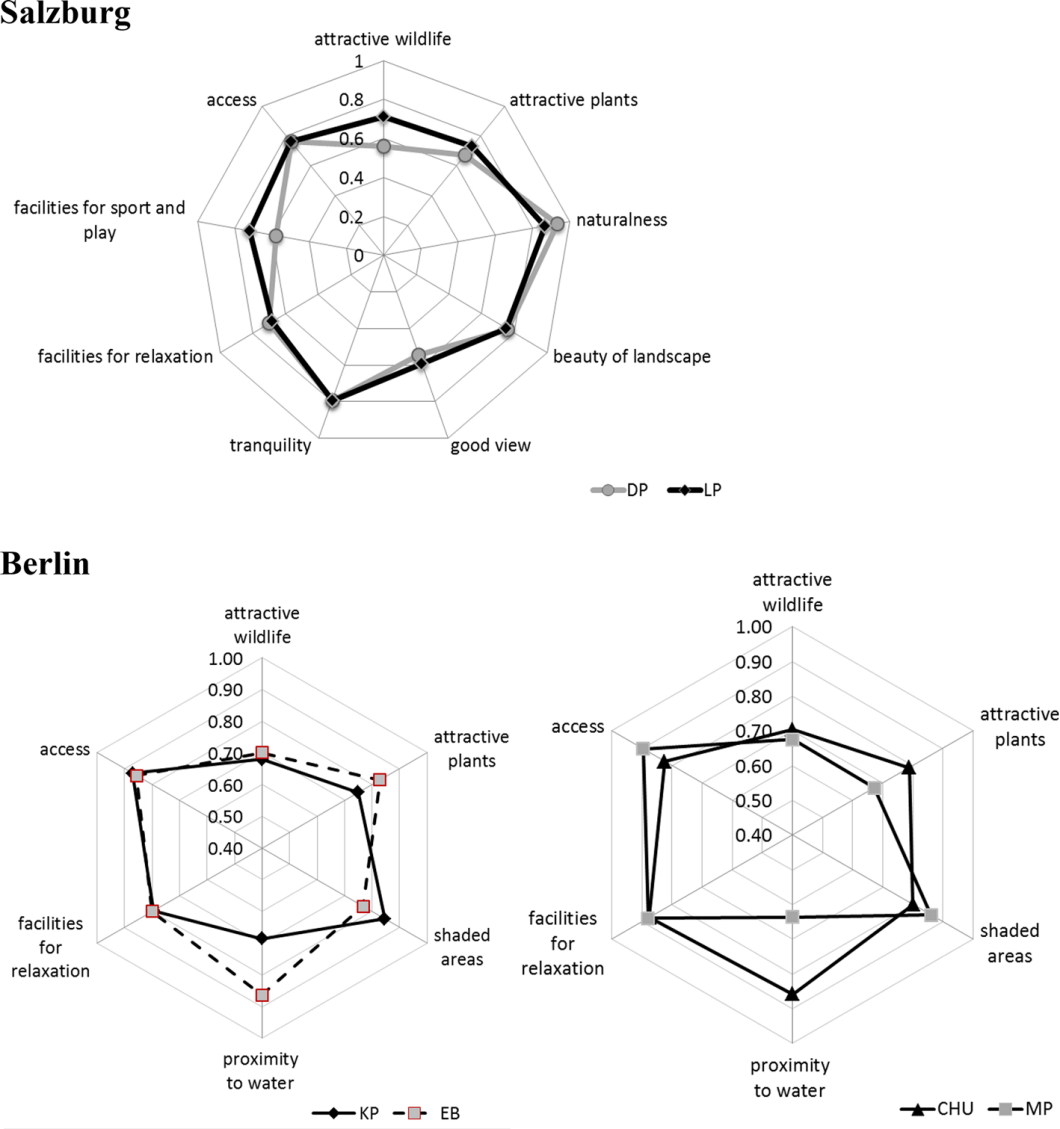
Assessment of importance of park characteristics by visitors in Salzburg and Berlin—with 0 representing not important to 1 representing very important (DP: *n* = 30; LP: *n* = 37; EB: *n* = 50; CHU: *n* = 49; KP: *n* = 48; MP: *n* = 50)

In Salzburg, the results of this assessment are nearly identical in both parks (Fig. 4). The most important characteristics are naturalness, followed by tranquility, access, and landscape beauty, and then park facilities for passive relaxation and for sports and play. Visitors of LP placed higher importance on sport facilities and playgrounds, plants, and wildlife than visitors of DP. In Berlin, where we asked in a slightly different way, the most important aspect for park visitors is the accessibility of the park followed by lawns as facilities for relaxation and shade areas. In EB and CHU, each of which contains a water element, the importance of water is higher than in all other characteristics. Further, in the highly managed EB, plants and flowerbeds are also considered important.

## Discussion

### Importance of Specific Park Characteristics and User Activities in Relation to Structural Diversity

In the following, we discuss the results of the questionnaire surveys against those of the park mappings to assess whether and how specific park users’ activities and their evaluation of the importance of park characteristics are related to the parks’ structural diversity. In doing so, Table 2 shows the highest scorings of the diversity assessment by using “−” and “+” to simply illustrate if the values are comparatively lower or higher than the overall mean value for all parks in the respective category. Further, Table 2 highlights in bold those park characteristics rated most important for visitors as well as the distribution of visitors’ activities differentiated into active and passive.

In the smaller parks in Berlin, visitors rated facilities for relaxation and shaded areas as most important. This is especially the case in KP and MP where biotic features such as large and old trees are able to provide shade. Proximity to water is highly valued in those parks, where water elements exist and therefore abiotic structures gained higher scores. This is especially the case for the CHU along the canal (+++) but also for EB with its large water basin although with lower rating (±). The EB is a highly managed and designed park but without groups of trees and, thus, not much shaded space. Biotic features are, thus, lower (compared to the general mean presented as “−”). But there are well-manicured flowerbeds and hedges which are obviously appreciated by visitors (high importance for attractive plants with 0.83). In conclusion, specific diversity structures of parks in Berlin are recognized and valued because of their attractiveness (e.g., flowerbeds) but also because of their mitigating advantages such as providing shade. These characteristics might be reflected in the activities of park visitors. In all parks in Berlin, physical activities were only marginally represented (again, Table 2). Visitors rather relax than actively engage in sports with only a slight difference at the CHU. The reason for the slightly higher values for physical activities in CHU might be that it is part of a greenway accompanying an urban canal and therefore is attractive for jogging. Further, the MP is the only park of the four assessed which contains specific designated sport areas but is not represented in the activity results. The low value of active exercise in Berlin might be explained by the smaller size of the parks and the lack of infrastructure for physical activities.

Interestingly in Salzburg, the most important park characteristics are more or less the same although the two parks have a different structural diversity. The DP scores very high for biotic features (+++) and active infrastructure (+++) while LP scores very high for passive infrastructure (+++). The different structural diversity values for infrastructure are represented in the activity assessment; in DP, we recorded more active recreational activities than passive activities (75 % compared to 25 %). However, when divided into group and single activities, single activities far exceed group activities (such as group sports and games). The scenic DP with its multiplicity of biotic features and a beautiful view to the Alps may encourage solitary activities such as walking or other activities for enjoying silence or nature. In addition, the dog park attracts people walking their dogs. By contrast, passive activities for recreation are more prominent in the LP. However, active group activities are also important while single activities are marginally represented. Obviously, the LP with a high number of infrastructures for active recreation attracts people who like to play or engage in sports with others. In addition, the small number of biotic features combined with an open and good all round visibility allows people to watch other people (and be watched).

Nevertheless, other (side) effects need to be considered when explaining park activities by visitors: the DP (7 ha) is a comparatively large park and is located within a less densely built-up residential area, partially surrounded by areas used for urban agriculture and recreation. As a part of an attractive green corridor, the DP may be an attractive component for a longer walk, bike, or jogging course. In contrast, the LP and most of the assessed parks in Berlin are smaller and located within densely built-up districts, often with no nearby park alternatives. It becomes apparent that parameters such as area size, recreational alternatives, and connectivity with other urban green structures have to be considered as well when interpreting the differences among park uses. In addition, more people with low income and more foreign nationals live in the densely built-up areas in Berlin as well as in the surroundings of LP than in the area of DP. Accommodation size, access to private green areas, and differences in social interactions, habits, and preferences due to cultural background (e.g., Rishbeth 2001) may have an influence on urban parks use as well.

In conclusion, the surveys in both cities revealed that specific properties are very important to park visitors and their activities. They can, in part, be linked to the three dimensions of structural diversity such as naturalness and the various facilities for active and passive recreation. However, not all park visitor preferences can be directly linked to these three dimensions of structural diversity. Accessibility is highly ranked by visitors in all parks, regardless of size or location in Berlin or Salzburg. Thus, structural diversity might not be the key factor for the entire recreational service of a green space, but one key factor amongst others.

### Methodological Issues

We introduced an integrative method that combines multi-dimensional mapping of urban parks’ diversity with users’ activities and their demand on urban park characteristics. This combination allows integrating objective and subjective data, that is, the urban parks’ recreational supply with the visitors’ demand. As result of the application in Berlin and Salzburg, the method resulted in being highly recommendable for park comparisons. The presented approach of structural diversity mapping is practicable and not time-consuming and links the man-made facilities and amenities with the biotic and abiotic features. The linkage with the interviews allows interpreting the various structures in light of their contributions to the provision of various recreational services.

Further, we only considered the absolute existence of particular elements, e.g., *old trees*, but not the number (of trees). Adding the number would certainly lead to more complexities. In addition, some atmospheric characteristics such as the dominance of specific user groups or disturbances in the park may be added to a possible follow-up study.

Although the differentiation of categories and the selection of elements for the mapping are based on an extensive literature review, there still remains a considerable degree of subjectivity. Nevertheless, we tried to reach the same level of details in the three structural diversity dimensions, but there is no objective measurement defining the “same” level. Since the specific differentiation and selection of elements is worth discussing, the method implies a very good adaptability to different urban parks and cities world-wide. It is intended that the catalog of diversity elements is changed and varied by adding or omitting elements according to requirements and conditions. Hence, it is easy to regard the specific characteristics of every urban park considered. This also applies for parks already investigated by questionnaire surveys and allows easily integrating existing data into the system for structural diversity.

Finally, the grouping of users’ activities into physical active exercises and passive relaxation resulted in being very fruitful as the distinction between group and single made in Salzburg also yields interesting differentiations. Also the data from the additional observation protocols helps a lot to consider activities from people who refused to participate in the survey due to participation in sports, group activities or playing with children (active recreation) or because of deficient language skills.

## Conclusion

Today, with a more and more diverse society, urban parks should meet a variety of different interests and demands and therefore need to be designed accordingly. An important requirement for providing a high multitude of alternatives of activities and enjoyment is certainly the size of the park, but we also have to regard the diversity of biotic and abiotic features as well as man-made facilities for sport and relaxation. Certain biotic features are appreciated such as large trees for shade while water elements seem to be important especially in the cases in which they have visual dominance. However, as our results show, other properties such as accessibility or tranquility are also very important.

The concept of linking the mapping of structural diversity with questionnaires is easy and straightforward to apply and can be adopted easily. By adding or omitting structural diversity elements, it is easy to consider the specific characteristics of every urban park surveyed. In the context of planning and developing urban green, this method provides an instrument for guidance to efficiently plan and manage urban green areas. Some important goals of urban park management are to provide natural and man-made features, facilities, and amenities that allow visitors to have satisfying recreational experiences. Meeting these goals is not easy; especially in urban areas where space is limited, demand on and use of green space is high, and demands, desires, and activities are very diverse. However, we have to regard both the various users’ desires, perceptions, and experience as well as the physical characteristics of the parks.

The proposed integrative method links the demand and the supply of recreational services of urban parks in a nuanced way. The ecosystem service approach assumes that ecosystem functions and biotic elements provide the basis for the supply of cultural services, but infrastructural facilities and amenities have a large impact on the recreational value of an urban park. Overall, we have to consider the perception, valuation and use by the visitors that may differ a lot depending on socio-cultural background. In this way, the ecosystem service approach has to be more comprehensive.
